# OP38 Policy Perspective On Next-Generation Precision Health In The New Zealand Health System

**DOI:** 10.1017/S0266462325100962

**Published:** 2025-12-29

**Authors:** Thomas Wilkinson, Alex Williams, James Oughton

## Abstract

**Introduction:**

The New Zealand health system provides universal health coverage to a population of 5.2 million, delivered through a range of public, private, and voluntary providers. New Zealand has extensive experience applying health technology assessment (HTA) to pharmaceuticals and vaccines. These models could potentially be adapted and applied to innovative precision health technologies that offer substantive potential health and equity benefits for patients and whānau (families), and health system financial sustainability.

**Methods:**

The New Zealand Ministry of Health developed a Long-Term Insights Briefing on Precision Health to highlight future trends, risks, and opportunities represented by genomics and artificial intelligence-enabled technologies. The briefing was developed collaboratively with consultation with patients and the public, including deliberative consultation with Māori and Pacific peoples. Organizational input was provided from across the sector including from government agencies, clinicians, researchers, industry, and patient organizations. The briefing was structured around key themes identified through engagement.

**Results:**

The briefing identified multiple themes with important implications for any future design and approach to the conduct and delivery of HTA for genomic and artificial-intelligence-enabled technologies in New Zealand. Core issues raised by stakeholders included the need for sustained and coordinated investment pathways, societal-wide consideration of the impact of new technology adoption, specific incorporation of multiple dimensions of equity, and consideration of impacts on the efficiency and financial sustainability of the health system. The importance of maintaining data sovereignty was a major element of the briefing, including the conduct of research and collection and use of information.

**Conclusions:**

This policymaker-led session detailed the incremental progress New Zealand has made toward realizing and managing the opportunities and risks of precision health. HTA processes and methodology are useful tools that may be adapted to ensure appropriate consideration of identified objectives and trade-offs associated with expanded investment and use of precision health technologies in New Zealand’s publicly funded health system.

